# Molecular phylogenetics reveals convergent evolution in lower Congo River spiny eels

**DOI:** 10.1186/s12862-015-0507-x

**Published:** 2015-10-15

**Authors:** S. Elizabeth Alter, Bianca Brown, Melanie L. J. Stiassny

**Affiliations:** Department of Biology, York College, City University of New York, 94-20 Guy R. Brewer Blvd, Jamaica, NY 11415 USA; CUNY Graduate Center, 365 Fifth Avenue, New York, NY 10016 USA; Sackler Institute for Comparative Genomics, American Museum of Natural History, 79th St and Central Park West, New York, NY 10024 USA; Department of Ichthyology, American Museum of Natural History, 79th St and Central Park West, New York, NY 10024 USA

**Keywords:** Phenotypic convergence, Cryptophthalmia, Fish, Congo River, Phylogenetics, Biogeography, Molecular divergence

## Abstract

**Background:**

The lower Congo River (LCR) is a region of exceptional species diversity and endemism in the Congo basin, including numerous species of spiny eels (genus *Mastacembelus*). Four of these exhibit distinctive phenotypes characterized by greatly reduced optic globes deeply embedded into the head (cryptophthalmia) and reduced (or absent) melanin pigmentation, among other characteristics. A strikingly similar cryptophthalmic phenotype is also found in members of a number of unrelated fish families, strongly suggesting the possibility of convergent evolution. However, little is known about the evolutionary processes that shaped diversification in LCR *Mastacembelus*, their biogeographic origins, or when colonization of the LCR occurred.

**Methods:**

We sequenced mitochondrial and nuclear genes from *Mastacembelus* species collected in the lower Congo River, and compared them with other African species and Asian representatives as outgroups. We analyzed the sequence data using Maximum Likelihood and Bayesian phylogenetic inference.

**Results:**

Bayesian and Maximum Likelihood phylogenetic analyses, and Bayesian coalescent methods for species tree reconstruction, reveal that endemic LCR spiny eels derive from two independent origins, clearly demonstrating convergent evolution of the cryptophthalmic phenotype. *Mastacembelus crassus*, *M. aviceps*, and *M. simbi* form a clade, allied to species found in southern, eastern and central Africa. Unexpectedly, *M. brichardi* and *brachyrhinus* fall within a clade otherwise endemic to Lake Tanganikya (LT) ca. 1500 km east of the LCR. Divergence dating suggests the ages of these two clades of LCR endemics differ markedly. The age of the *crassus* group is estimated at ~4 Myr while colonization of the LCR by the *brichardi-brachyrhinus* progenitor was considerably more recent, dated at ~0.5 Myr.

**Conclusions:**

The phylogenetic framework of spiny eels presented here, the first to include LCR species, demonstrates that cryptophthalmia and associated traits evolved at least twice in *Mastacembelus*: once in *M. brichardi* and at least once in the *M. crassus* clade. Timing of diversification is broadly consistent with the onset of modern high-energy flow conditions in the LCR and with previous studies of endemic cichlids. The close genetic relationship between *M. brichardi* and *M. brachyrhinus* is particularly notable given the extreme difference in phenotype between these species, and additional work is needed to better understand the evolutionary history of diversification in this clade. The findings presented here demonstrate strong, multi-trait convergence in LCR spiny eels, suggesting that extreme selective pressures have shaped numerous phenotypic attributes of the endemic species of this region.

**Electronic supplementary material:**

The online version of this article (doi:10.1186/s12862-015-0507-x) contains supplementary material, which is available to authorized users.

## Background

**Fig. 1:**
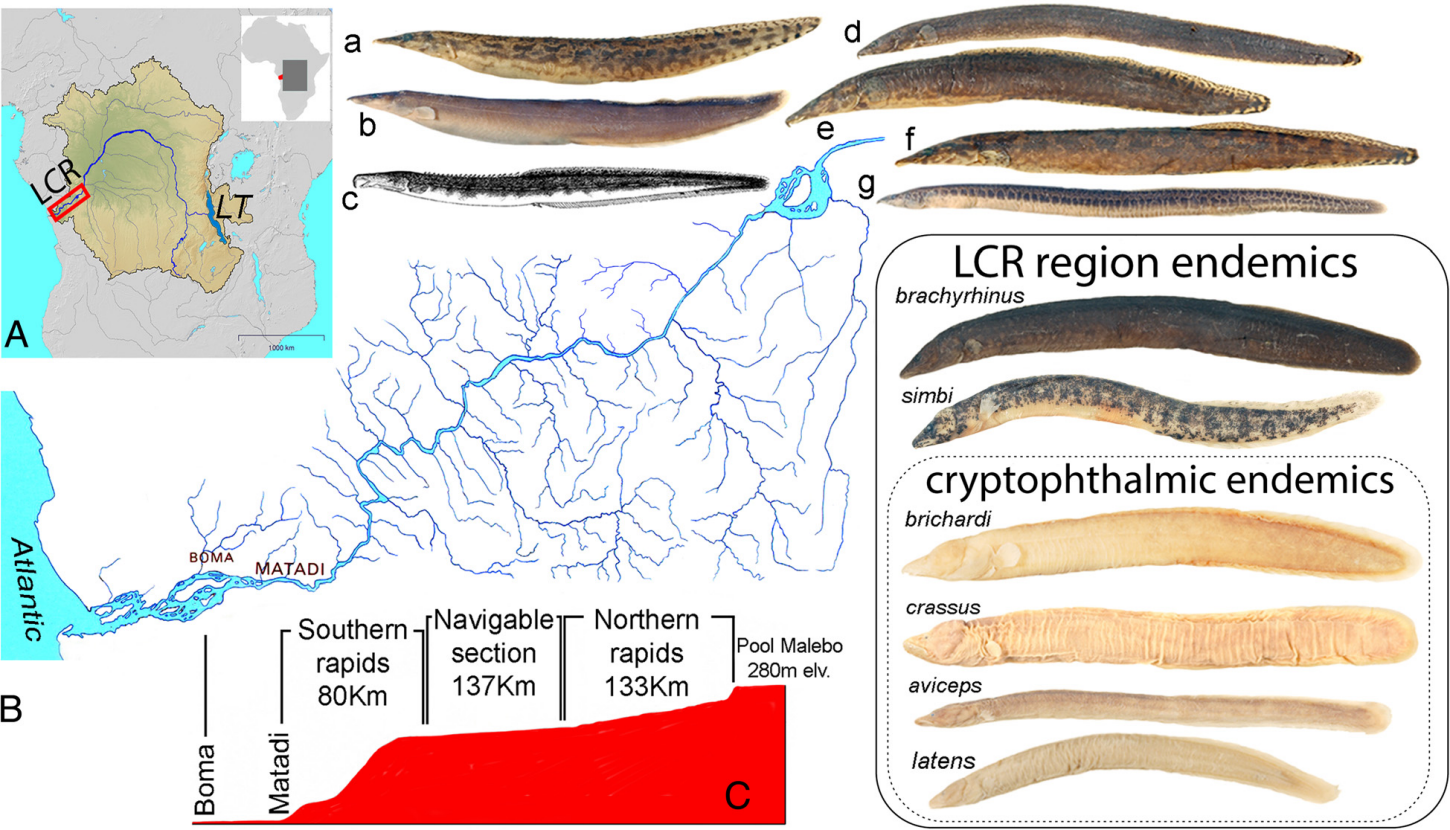
Mastacembelid diversity in the lower Congo River (LCR). **a** Location of LCR in the Congo basin. Main stem of the Congo River and Lake Tanganyika (LT) highlighted. **b** LCR and affluent tributaries from upstream Pool Malebo (upper right) to Estuary. **c** LCR elevational profile from Pool Malebo (280 m asl) to Boma at inner estuary. Native *Mastacembelus* species: a) *M. congicus*, b) *M. robertsi*, c) *M. traversi*, d) *M. niger*, e) *M. paucispinis*, f) *M. marchei*, g) *M. greshoffi*. LCR regional endemics: *M. brachyrhinus*, *M. simbi*, *M. brichardi*, *M. crassus*, *M. aviceps*, *M. latens*

**Fig. 2:**
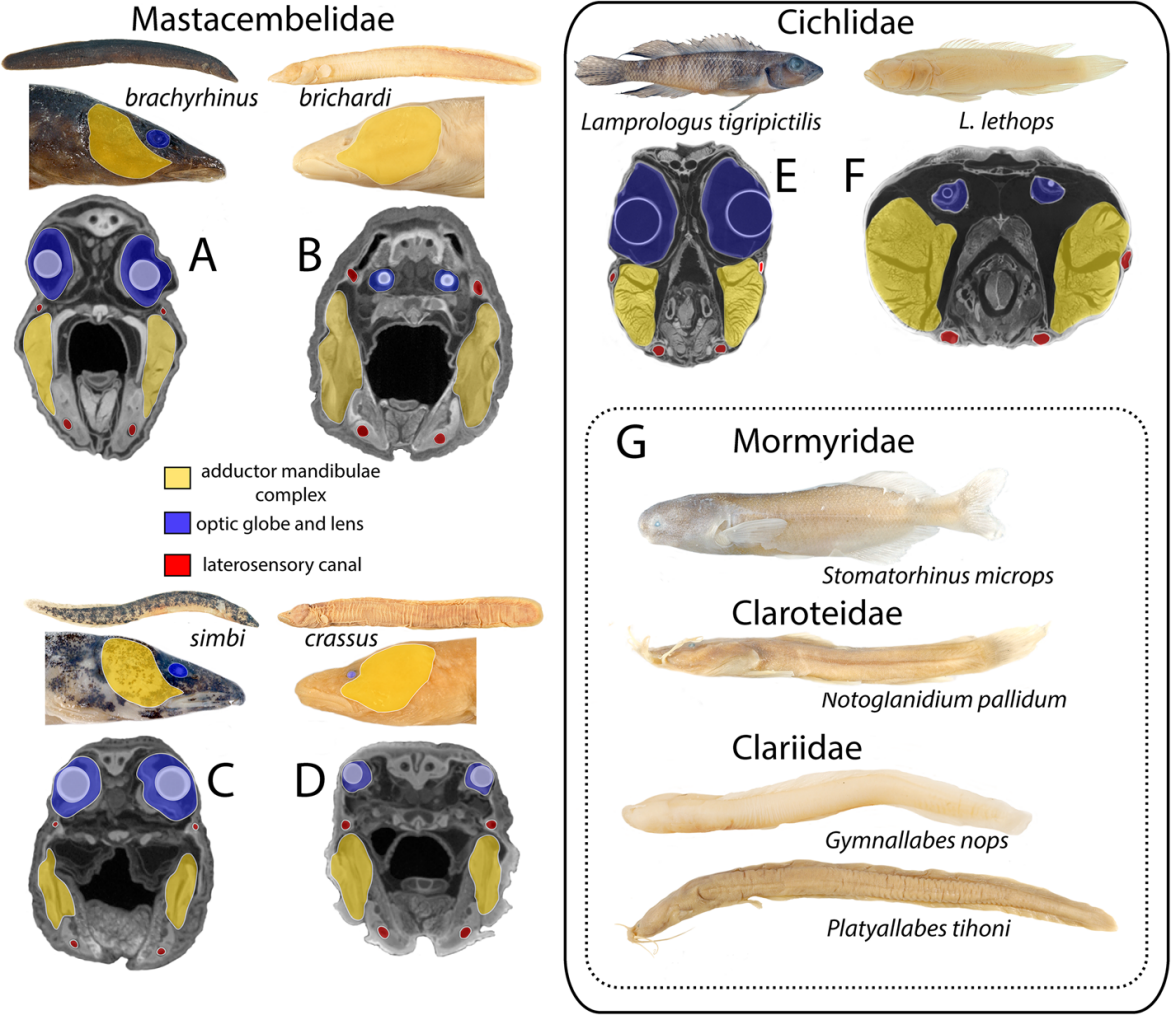
Cryptophthalmic phenotypes across LCR region endemics. **a**-**f** contrast pigmented and fully eyed and cryptophthalmic sister pairs. **g** Additional cryptophthalmic representatives from other fish families found in the LCR, demonstrating the taxonomic breadth across which this phenotype occurs

Recent estimates report the presence of 1269 species in the riverine network of the Congo basin, and of these 846 are regional endemics [1]. Despite occupying a little less than 2 % of the basin area, the lower Congo River (LCR) harbors a surprisingly high proportion of this diversity with a total of 328 recorded species, of which more than 80 are considered LCR endemics [2, 3]. Species endemism in the LCR spans numerous fish families but is particularly noteworthy among two, the Cichlidae (cichlids) and the Mastacembelidae (spiny eels). Although less speciose than cichlids, richness of mastacembelid spiny eels in the LCR is matched only by the better-known radiation of Lake Tanganyika (LT), some 1500 km to the east of the Congo basin (Fig. 1a). Currently 13 spiny eels have been reported from the LCR [4], and six of these are endemic to the system and immediate vicinity. Four exhibit strikingly distinctive phenotypes characterized by greatly reduced optic globes covered by skin and deeply embedded into the head (cryptophthalmia), reduced (or absent) melanin pigmentation, enhanced cephalic laterosensory systems, and hypertrophied jaw musculature (Fig. 2a-d), among other characters. However, little is known about the evolutionary history of LCR spiny eels, the timing of colonization of the LCR, or their biogeographic origins. In particular, it is not known whether the distinctive cryptophthalmic phenotype has a single origin, or has evolved convergently multiple times.

Examples of convergent evolution abound across natural systems (e.g. [5–8]), and have long generated important insights into the processes generating our planet’s diversity [9]. Phenotypic convergence is frequently taken as evidence of strong selective pressures [5–7], particularly in extreme environments such as caves [10, 11] and white sand ecosystems [12, 13]. However, convergence among closely related species may go unrecognized in the absence of robust molecular phylogenetic reconstructions (e.g. [14]). If a single origin is inferred for phenotypically similar taxa within a genus, for instance, the role of selection in generating morphological and species diversity may be underestimated [15, 16]. Inferring a phylogenetic framework based on molecular data, therefore, is a crucial first step in understanding the evolutionary processes underlying phenotypic convergence and species diversification.

In general, such evolutionary processes have been poorly studied in African rivers, where morphological diversity and endemism tend to be lower than in lacustrine systems [17, 18]. However, as noted above, the LCR represents a striking exception to this generalization, with high rates of endemism and morphological innovation across many fish families.

The LCR forms the outflow of the Congo River from Pool Malebo at an elevation of ca. 280 m above sea level which, over the course of ~350 km, descends in a series of stepped elevational drops to sea level (Fig. 1c). The Congo River drains roughly 3.8 million km^2^ of central Africa. On leaving Pool Malebo an annual discharge of 1250 x 10^9^ m^3^ of water plunges in a bedrock-constrained channel through intermittently narrow (<0.2 km) and wide (>2 km) gorges. In the process, this enormous volume of water forms a series of more than 60 major rapid systems and deep (>200 m) underwater canyons [19, 20]. In this relatively short but hydraulically complex stretch of river, more than 80 narrowly endemic fish species across numerous families have been identified [3]. While the underlying drivers of such high levels of species endemism in the region are not yet known, habitat heterogeneity, complex bathymetry, and extremely high-energy hydraulic conditions in the LCR have been hypothesized to play a major role in isolating populations, essentially resulting in allopatric speciation on extremely small geographic scales [21]. In addition to separating populations and promoting allopatric speciation, the numerous rapids systems and complex hydrology of the LCR also likely result in diversification via natural selection and adaptation, leading to unusual phenotypes observed across a broad phylogenetic spectrum of fish taxa [22]. In this regard it is noteworthy that, in addition to four mastacembelid species, the LCR harbors at least 5 additional endemic species from distantly related families that exhibit strikingly similar cryptophthalmic phenotypes (*Lamprologus lethops* (Cichlidae), *Stomatorhinus microps* (Mormyridae), *Notoglanidium pallidum* (Claroteidae), *Platyallabes tihoni* and *Gymnallabes nops* (Clariidae) (Fig. 2a-d).

The nature of the evolutionary processes generating these phenotypes is poorly understood, but such morphological convergence across unrelated lineages is highly suggestive of common selective pressures. While the phenotypic similarities between these LCR species and numerous hypogean, trogloditic species are striking, the environments that have shaped these characteristics appear to be quite different [23]. Most obviously, the LCR is not a lightless, still water environment. However, the main channel is extremely deep (>200 m) in some regions [20], and the presence of numerous crevices created by large boulders extending along alternately shallow and deep shoreline habitats, as well as high levels of suspended particulate matter and deep eddies, may create extremely low-light conditions in the system [24]. Although detailed knowledge of the precise microhabitat requirements for most cryptophthalmic LCR taxa is currently lacking, with the exception of the cichlid *Lamprologus lethops*, which appears to be a deep water, benthic species, most other cryptophthalmic forms have been collected in both shallow and deep waters, exclusively among rocks, and most often associated with rapid systems. In these rocky, high-energy, shoreline locations, which form the dominant habitat type along much of the LCR, cryptophthalmic individuals have been collected in sympatry with numerous non-cryptophthalmic taxa.

Spiny eels present an excellent case study in the context of speciation in the LCR, as the cryptophthalmic phenotype described above is present in multiple species. African mastacembelids are found in both rivers and lakes throughout the continent and number 50 described [25], and numerous undescribed [26, 27] species, but only two regions with high numbers of sympatric species have been identified: the LCR and Lake Tanganikya (LT). Monophyly of African mastacembelids is well supported in previous morphological studies [26, 28, 29], but prior to the study of Brown et al. [27] little phylogenetic resolution had been achieved beyond the suggestion that the LCR endemics formed a monophyletic assemblage possibly allied with certain members of the LT flock [4, 26, 28]. Brown et al’s study provided the first molecular analysis of relationships among mastacembelids with a focus on the phylogeny of LT spiny eels in the context of other African species [27]. However, no LCR representatives were included in that study.

Here, we augment the mitochondrial and nuclear dataset of Brown et al. [27] with the addition of genetic data from both native and endemic LCR region spiny eels and additional non-LCR congeners to provide a phylogenetic framework for LCR spiny eel diversification. In particular, we investigate the following questions: 1) Are endemic LCR species monophyletic within *Mastacembelus*, indicating a single colonization and single origin of the cryptophthalmic phenotype? 2) What are the phylogenetic relationships between spiny eels in the LCR and other biogeographic regions in Africa? 3) When did spiny eels colonize and diversify in the LCR? To address these questions, we used Bayesian and Maximum Likelihood approaches, including Bayesian coalescent methods for species tree reconstruction, to determine the evolutionary relationships among LCR endemics and evaluate their phylogenetic position within the context of the African Mastacembelidae.

## Methods

### Sample collection

Samples were collected in the Democratic Republic of Congo (DRC) and Republic of Congo (RC) between 2006–2015 from sites across the LCR, Pool Malebo, and middle Congo River. Fishes were collected and euthanized prior to preservation in accordance with recommended guidelines for the use of fishes in research [30]. Tissue samples were stored in 95 % ethanol, and voucher specimens cataloged into the ichthyology collection of the American Museum of Natural History (AMNH), available online at (http://sci-web-001.amnh.org/db/emuwebamnh/index.php). GenBank, museum catalog and tissue numbers, as well as collection data, are given in Additional file 1: Table S1. In addition to LCR species available for sampling (*congicus*, *robertsi*, *niger*, *paucispinnis*, *greshoffi*, *brachyrhinus*, *brichardi*, *simbi*, *crassus*, *aviceps*), we included tissues from 5 other *Mastacembelus* species (*seiteri*, *cryptacanthus*, *liberiensis*, *taiaensis,* and *M*. sp. “Lulua-Tshikapa”) represented in AMNH collections. To extend geographical representation and taxonomic coverage, additional sequences from Brown et al. [27] were obtained from GenBank.

### DNA extraction, amplification and sequencing

DNA was extracted from tissue preserved in ethanol using the Qiagen DNeasy kit (Qiagen Inc). We amplified the cytochrome *b* marker using primers MNCN-GLUF [31] and MNCN-ProR [27] with the following amplification protocol: 35 cycles of 3 min 94 °C for initial denaturation, with subsequent cycles of 30s/94°C, 30s annealing at 48 °C, 1 min/72 °C extension, with a final 5 min extension at 72 °C. Amplifications were performed in 25 ul reactions using Illustra PCR beads (GE Healthcare) and 10–20 ng of genomic DNA. Two nuclear introns, the first and second introns of the ribosomal protein S7 gene (S7RP1 and S7RP2) using published primers [32] and the same PCR profile as above were also amplified. These markers were chosen to maximize comparisons with published and available data from Brown et al. [27] – additional studies using more slowly evolving markers such as RAG1 are ongoing (Day et al. in prep). S7 is a single copy nuclear gene in teleosts with relatively low within-species variation, and the first two introns within this gene have proven to be useful in examining relationships within families (e.g. [33]). Successful amplifications were cleaned using an Exo-SAPit protocol (Amersham Biosciences) and sequenced on an ABI 3730 XL (Applied Biosystems) in the Sackler Institute for Comparative Genomics, AMNH.

### Alignment and phylogenetic analysis

Contig assembly and sequence editing were performed using Geneious Pro v6.1.4 (Biomatters, available from http://www.geneious.com/). Each gene partition was aligned using MUSCLE [34]. In addition to the samples sequenced here, the dataset was aligned with *Mastacembelus* sequences from a previous study [27]. The best model of evolution for each marker was determined using the Findmodel web application (http:http://www.hiv.lanl.gov/content/sequence/findmodel/findmodel.html). Application of Akaike information criterion across 12 possible models indicated that the best fitting model for cyt *b* was GTR + G + I and for the two introns, HKY. Arlequin v.3.0 [35] was used to assess *F*_*ST*_ and genetic distance for two species (*M. brichardi* and *M. simbi*) for which we had multiple individuals from two distinct locales in the LCR region (upstream and downstream of Pool Malebo).

Using the aligned datasets, phylogenetic relationships were estimated applying three optimality criteria. First, Maximum Likelihood (ML) inference was performed on the concatenated dataset using RAxML 8.0 [36]. Results from Findmodel indicated a GTR + G + I model for the first partition (cyt *b*) and HKY for the second (S7 introns); as RAxML does not implement HKY, the GTRGAMMA model incorporating rate heterogeneity was used for both. The cyt *b* gene was also partitioned by codon position. A rapid bootstrap analysis with 500 bootstrap replicates was used to assess branch support. Second, Bayesian inference (BI) was performed in BEAST v1.7.5 [37] on the concatenated dataset, using Markov Chain Monte Carlo simulation. BEAST was run with a Yule model as the tree prior with unlinked substitution and clock models and used two independent runs with 50 million generations each (sampling every 1,000 generations, four chains including one cold and three heated (temperature = 0.1) with burn-in of 1 % (xml file available as Additional file 2: Supplemental Information file 1). These analyses were also performed on individual alignments of mitochondrial and nuclear loci (cyt *b* and concatenated S7 introns). Third, STAR-BEAST v.2.1.3 [38] was used to perform multispecies coalescent Bayesian inference of tree topology, using an uncorrelated lognormal relaxed molecular clock and a Yule prior on speciation rate. Tracer v. 1.4 [39] was used to assess adequacy of burn-in and model convergence for Bayesian analyses. Two Asian species, *Mastacembelus armatus* and *Macrognathus zebrinus*, were designated as outgroups in all analyses.

### Specimen designations and taxonomic considerations

Based on preliminary results from BI and ML analyses, the single individual of *M. brachyrhinus* (SA77712, Baidou River, Ubangi basin, Central African Republic) from Brown et al. (2010) [27] was reassigned here as *M*. sp.“CAR” as it did not group with the LCR samples of *M. brachyrhinus* introduced in this study and because the type locality of *M. brachyrhinus* is at Matadi in the lower reaches of the LCR (Fig. 1). We note, however, that two additional specimens from localities well outside of the LCR region have also been identified as *M. brachyrhinus*: MRAC 89-43-P-3632, collected in the Wela River, south of Kisangani [26] and AMNH 248082 also from near Kisangani [4]. Both of these specimens were collected in localities nearly 1800 river kilometers upstream from the most upstream collection site of *M. brachyrhinus* in the LCR (AMNH 246931, at Lenga Lenga near the RC border in the middle reaches of the LCR). Although no tissues of Kinsangani-region *M*. “*brachyrhinus*” are available for inclusion in the present study, we anticipate that, as for the Ubangi basin specimen (SA77712), individuals from this region likely belong to an undescribed taxon with no close affinity to *M. brachyrhinus.* Herein we consider *M. brachyrhinus* to be endemic to the middle and lower reaches of the LCR.

Similarly, specimens in the AMNH collections from the Lulua River and vicinity of Tshikapa (upper Kasai basin, DRC), although initially identified as *M. congicus,* group with the anomalous M.sp.”CAR” specimen rather than with the other *M. congicus* specimens included in this study. As currently recognized *M. congicus* is an extremely widespread species found throughout the Congo basin, but based on results here, is likely a cryptic species complex the resolution of which will require intensive sampling from across the range of the nominate species. For this reason we have designated the Lulua-Tshikapa specimens as *M*.sp. “Lulua-Tshikapa”, and retained the name *M. signatus* for specimens from Lake Bangwelu and the Chambeshi River despite the recent synonomy of that species with *M. congicus* [40]. Such cryptic diversity is likely also to be the case for other “widespread” species, such as *M. frenatus,* and more extensive sampling than that undertaken here will be necessary to fully resolve the species composition and biogeographic histories of these putatively widespread taxa (Day et al., in prep).

### Estimation of divergence times

Divergence times were estimated using a relaxed-clock method with substitution rates sampled from an uncorrelated lognormal distribution implemented in BEAST v. 2.1.3 [37]. Calibration remains a major challenge for this group, because no fossil record is available for any mastacembelid or synbranchiform taxon. Previous analyses have used external fossil calibration points from the Channidae (snakeheads) based on their close relationship with Synbranchiformes [41]. The oldest African fossil channid record, in the genus *Parachanna*, is dated to the late Eocene (33–35 Myr based on paleomagnetic dating) from Egypt [42]. The Channidae includes two genera, *Channa*, distributed in Asia, and *Parachanna*, with three species endemic to Africa. Consequently we followed the method of [27] and used the fossil calibration of 33–35 Myr for the split between the genera *Channa*-*Parachanna* as a monophyletic stem group (as the fossil *Parachanna* was not assigned to any modern species). All other ages were estimated relative to the *Channa* + *Parachanna* node. Sequences for representatives of taxa were obtained from NCBI/GenBank (Additional file 1: Table S1). Because S7 intron data are not available for *Channa-Parachanna* representatives, only cytochrome *b* data were used in divergence date analysis, and therefore it was run as a separate analysis from the Bayesian inference on the concatenated dataset described above. We also attempted to use the estimated range of divergence times for two nodes within the Percomorpha based on multiple fossil calibration points following [43], but this resulted in heavy saturation at cytochrome *b*, causing homoplasies that produced unreliable phylogenetic relationships. To account for uncertainty in the date estimates, we constrained the *Channa*-*Parachanna* node with a zero-offset of 34 million years, and applied a log-normal mean of 0.01 and log-normal standard deviation of 1.5, giving a median age of 34.0 million years and 97.5 % prior credible interval extending to the lower Eocene, 52.1 million years (xml file available in Additional file 2: Supplemental Information file 2). Two independent MCMC simulations with 100 million iterations (sampling every 1,000 generations) were performed. Runs were combined and checked for convergence in Tracer v. 1.4 [39]. TreeAnnotator [44] was used to calculate the maximum clade credibility tree with burn-in of 1 %.

## Results and discussion

**Fig. 3:**
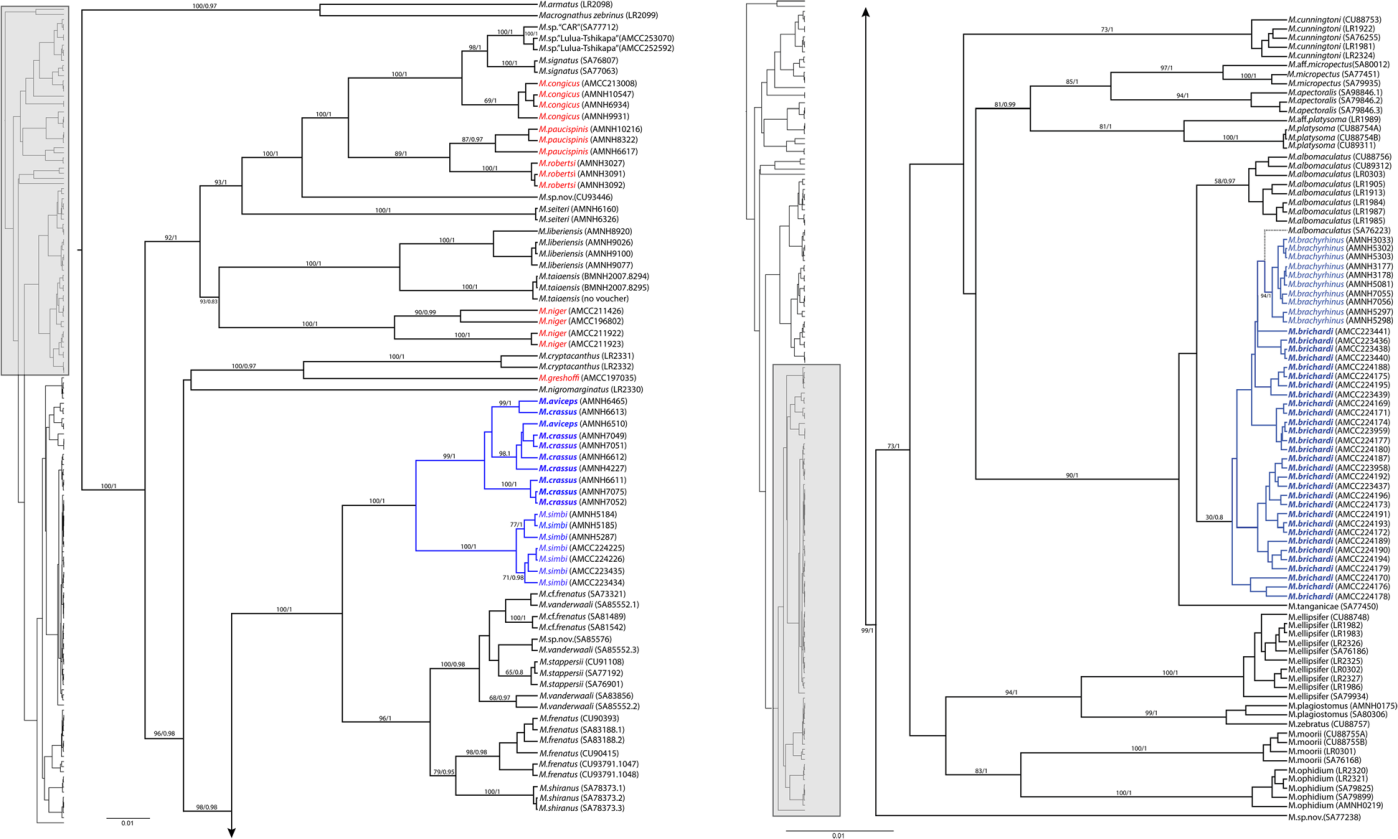
Phylogenetic trees constructed from (i) BI and (ii) ML methods using the full concatenated dataset. Branch lengths are taken from BI and are proportional to substitutions per site. Node values represent Bayesian posterior probabilities (above branch) and bootstrap values (below branch). Names in blue italic indicate endemic non-cryptophthalmic LCR (Lower Congo River) species, names in blue bold italic indicate endemic cryptophthalmic LCR species, names in red italic indicate native, non-cryptophthalmic LCR species

**Fig. 4:**
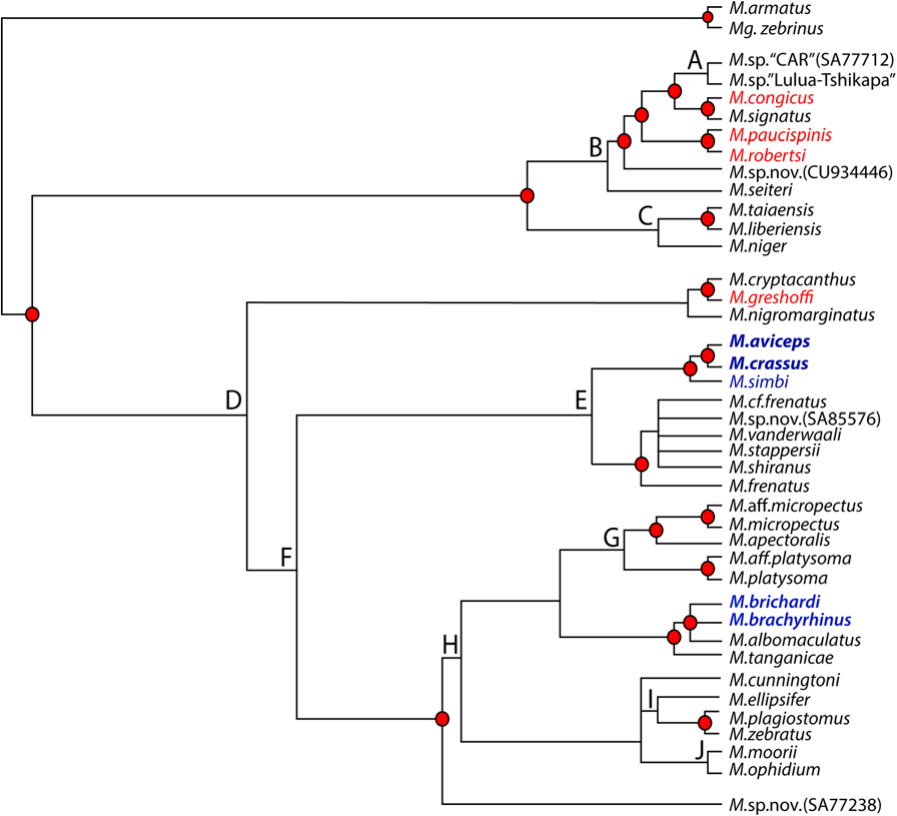
Species tree of African mastacembelid eels inferred from mitochondrial (cyt *b*) and two nuclear (S7 introns) markers (2702 bp), using coalescent-based species tree analysis with no data concatenation (STAR-BEAST). Nodes with Bayesian posterior probabilities >0.98 are indicated by circles at nodes. Letters correspond to node support values in Table 1 (nodes with posterior probabilities <0.6 collapsed). Names in blue italic indicate endemic non-cryptophthalmic LCR (Lower Congo River) species, names in blue bold italic indicate endemic cryptophthalmic LCR species, names in red italic indicate native, non-cryptophthalmic LCR species. Two Asian species (*Mastacembelus armatus* and *Macrognathus zebrinus*) are used as outgroups

After editing and trimming, the final dataset consisted of a total of 2495 base pairs aligned across three markers (cytochrome *b* and two S7 introns) for 157 individuals (with 80 individuals included from [27]). The cytochrome *b* marker was 1199 bp (including 1119 bp cytochrome *b* and 80 bp flanking tRNA genes) and the alignment did not contain any indels. The first s7 intron had an aligned length of 560 bp including flanking coding sequence, and second intron had an aligned length of 736 bp including flanking coding sequence; both introns contained non-contiguous indels ranging in size from 1-17 bp. Data were missing for one specimen for cytochrome *b* and for three specimens for the first S7 intron (Additional file 1: Table S1). Our complete dataset includes representatives of 37 species, comprising upward of 70 % of the described diversity of African *Mastacembelus*. Coalescent-based species tree and ML and BI phylogenetic reconstructions on the concatenated dataset were generally well resolved, with near identical tree topologies at supported nodes (Figs. 3, 4, Additional file 3: Figure S1 and S2). While the majority of species designations were well-supported (25 of 30 for which multiple specimens were available), discordance between mitochondrial and nuclear results was observed in the case of *M. crassus* and *M. aviceps*, and two instances in which specimens of a designated species did not form a single supported clade (*M. albomaculatus* and *M. vanderwaali*) were found.

### Multiple colonizations of the LCR

Regardless of the method used, all phylogenetic analyses indicate strong support for two independent origins of endemic LCR mastacembelid eels, which fall into two distinct and well-supported clades (Figs. 3 and 4). *M. brachyrhinus* and *M. brichardi*, are nested within a clade otherwise composed of species endemic to Lake Tanganikya (LT). Interestingly, the close relationship between the *brachyrhinus*/*brichardi* and LT species, particularly *M. albomaculatus* and *M. tanganicae,* mirrors a similar biogeographic pattern observed in lamprologine cichlids [45, 46]. It has been suggested that tectonic changes during the Neogene reconfigured drainage systems across eastern and central Africa, creating an ancient river system (the proto-Malagarasi) that drained west into the Congo River [47]. It has also been hypothesized that adaptation to high-energy riverine habitats may have served to “preadapt” such rheophilic species for subsequent colonization of rocky shoreline habitats of LT and other lakes as these were formed later in the Neogene [21, 22]. However, the phylogenetic results presented here suggest a possible reverse scenario in which progenitors of the LCR radiation, adapted to the rocky habitats and deep waters of LT, gained access to the Congo River and its major tributaries long after the formation of deep water conditions in the lake. On dispersing through the rivers of the basin, the ancestors of modern LCR species, already preadapted to occupation of deep, rocky habitats, were able to colonize the bedrock constrained, rocky shore lines and deep waters of the LCR and to radiate there.

The other LCR region endemics, *M. crassus*, *M. aviceps*, and *M. simbi*, are resolved as sister to a well-supported clade that is widely distributed across southern, eastern and central Africa. A fourth, cryptophthamic endemic, *M. latens* (Fig. 1) is currently known only from the formalin-fixed holotype and two paratypes, and despite considerable collecting efforts at the type locality of Bulu in the middle reaches of the LCR, no additional specimens have yet been obtained. However, morphological examination of the type series indicates unambiguously that *M. latens* is another cryptophthalmic member of the *crassus* clade, and is likely the sister species of *M. simbi* (Stiassny in prep.). Members of the sister clade to these LCR endemics include *Mastacembelus frenatus*, likely a species complex (Day et al., in prep.) that is widespread across eastern Africa and Congo and found in flood plains and marginal swamps but not in rapids or rocky habitats [28], *M. vanderwaali* found in the Okavango and Upper Zambezi system in rocky rapids [48], *M. stappersii*, which occurs in a small region of the Zambian Congo and occupies both benthic and pelagic habitats, and the Lake Malawi catchment endemic, *M. shiranus,* which occurs mainly in shallow waters in the lake often among rocks but is also found in swampy regions and among weeds in rivers [26]. Thus, members of the sister clade to the *M. crassus* group LCR endemics span a wide diversity of habitats and a broad geographical range. All are large bodied species (ranging between 400—284 mm maximum recorded total length) reflecting potentially significant dispersal abilities. In contrast, members of the *M. crassus* complex are geographically highly localized, stenotypic, and found exclusively in shoreline rocky habitats, often at depth and usually associated with rapids in the LCR region. All are notably small-bodied species; the largest being *M. crassus* (max. 164 mm) and the smallest *M. latens* (max. 72 mm) and *M. simbi* (max. 78 mm), the latter two being the smallest known *Mastacembelus* species. A similar, broadly Congo-Zambezian phylogeographic affinity has been demonstrated for a number of other fish groups including mochokid catfishes, serranochromine cichlids, and the alestid genus *Hydrocynus* (e.g., [49–52]) but none of these studies focused on LCR taxa. A more through sampling of putative mastacembelid species from this large and biogeographically complex region is needed to begin to unravel the biogeographic history and possible colonization scenarios for this clade of LCR mastacembelids (Day et al. in prep).

### Independent origin of cryptophthalmic phenotypes

Our results clearly indicate two independent origins of the cryptophthalmic phenotype among endemic LCR *Mastacembelus* (Figs. 3 and 4). Many aspects of this divergent phenotype are also shared across other distantly related fish lineages found in the LCR (Fig. 2). For example, the suite of features exhibited by the cryptophthalmic cichlid, *Lamprologus lethops* (Fig. 2g) as compared with its sympatric sister species, *L. tigripictilis* (Fig. 2f) is remarkably similar to those exhibited by the two phylogenetically divergent mastacembelid sympatric species pairs, *Mastacembelus brachyrhinus* (Fig. 2a) and *M. brichardi* (Fig. 2b), and *M. simbi* (Fig. 2c) and *M. crassus* (Fig. 2d). We hypothesize that the selective environment imposed by the LCR’s unique bathymetry and hydrology has shaped the evolution of these distinctive phenotypes multiple times and across phylogenetically disparate fish families. Such observations underscore the potential of the LCR as a biogeographic locus for future studies across a broad phylogenetic spectrum, using genome-wide markers combined with detailed morphological analyses to explore the mechanisms underlying such iterative phenotypic diversification (Alter and Stiassny in prep.).

### Divergence dating and the age of the lower Congo rapids

**Fig. 5:**
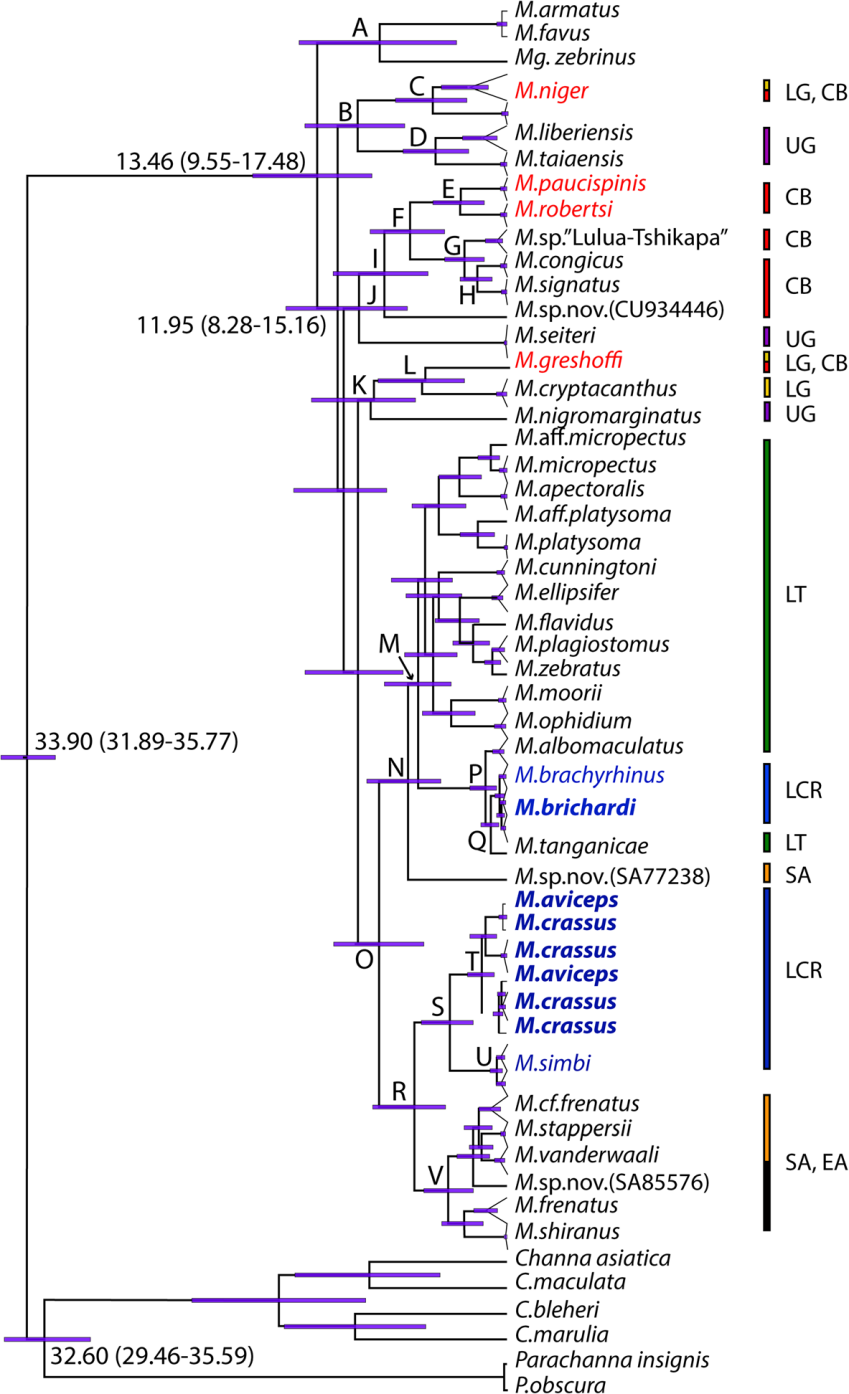
Time-calibrated phylogeny of spiny eels inferred from cytochrome b data using Bayesian inference (BEAST) with a relaxed clock. Letters correspond to divergence date values (MRCA mean, highest posterior density values) in Table 2. Names in blue italic indicate endemic non-cryptophthalmic LCR (Lower Congo River) species, names in blue bold italic indicate endemic cryptophthalmic LCR species, names in red italic indicate native, non-cryptophthalmic LCR species. Geographical abbreviations: CB, Congo Basin; EA, eastern Africa; LCR, Lower Congo River; LG, Lower Guinea; LT, Lake Tanganyika; SA, southern Africa; UG, Upper Guinea

Bayesian analysis of divergence dates indicates that the origins of the LCR endemic clades are of markedly different ages. While the date of the most recent common ancestor for each clade does not necessarily correspond exactly to the date of colonization, it does place a boundary on the earliest point at which colonization could have occurred in each group. These estimates indicate that the ancestor of the *M. crassus* complex (MRCA mean = 4.04 Myr, 95 % HPD [highest posterior distribution] = 2.43-6.06 Myr) was already present in the LCR long before the colonization of the system by *M. brichardi* and *M. brachyrhinus* (0.51, 95 % HPD = 0.26-0.91 Myr) (Fig. 5, Table 2). The estimated ages of modern species likewise differ between clades but fall in the late Neogene/early Pleistocene (*crassus* and *aviceps* 1.78, 95 % HPD: 0.97–2.79 Myr; *simbi* 0.74, 95 % HPD: 0.32–1.21; *brichardi* 0.4, 95 % HPD: 0.32–1.36; *brachyrhinus* 0.29, 95 % HPD: 0.15–0.94). Estimated time of colonization for the LT clade (7.02, 95 % HPD: 4.70–9.90 Myr) is similar to an earlier study which found a 95 % HPD for this clade of 5.5–10.6 Myr [27], concordant with, or slightly older than, estimated ages for the formation of the LT basins (9–12 Myr old [53] and 5.5 Myr or younger [54]).

**Table 1:** Posterior support values for species tree shown in Fig. 4, corresponding to lettered nodes

A	0.90
B	0.89
C	0.70
D	0.92
E	0.87
F	0.87
G	0.60
H	0.67
I	0.97
J	0.87

**Table 2:** Divergence estimates in millions of years (Myr) based on results of Bayesian molecular dating using BEAST v2.1.3 (corresponding to lettered nodes in Fig. 4)

Node	Divergence time (Myr)
	Mean (95 % CI)
A	9.01 (3.58–14.7)
B	10.58 (7.25–14.27)
C	5.26 (2.85–7.89)
D	5.06 (2.77–7.35)
E	3.24 (1.59–5.22)
F	6.86 (4.43–9.69)
G	2.98 (1.66–4.43)
H	2.12 (1.12–3.33)
I	8.68 (5.63–12.29)
J	11.95 (7.10–14.28)
K	9.54 (6.46–13.82)
L	5.98 (3.08–9.10)
M	6.27 (3.97–8.69)
N	7.02 (4.70–9.90)
O	9.05 (5.86–12.27)
P	1.58 (0.74–2.64)
Q	0.51 (0.26–0.91)
R	6.55 (4.39–9.56)
S	4.04 (2.43–6.06)
T	1.78 (0.97–2.79)
U	0.74 (0.32–1.21)
V	4.18 (2.37–6.89)

Understanding of the precise age and manner of formation of the present day Congo River network is uncertain, but considerable progress has been made in the past decade. Most recently [55] summarize a substantial body of evidence from terrigenous depositional studies, regional geomorphological analyses, and phylogeographic studies to reconstruct Neogene evolution of the basin. Regarding the evolutionary dynamics in the western basin, marine sediment studies from along the Atlantic coastal zone suggest intermittent outflow and a progressive southward migration, from the late Cretaceous through the mid Cenozoic, of the location of the main depocenter for terrigenous sediments from the internal Congo basin. Although a final consensus has yet to be reached, interpretation of palaeosedimentological data and analyses of present-day river topolology suggest that by the mid to late Miocene the main depocenter of sedimentation from the Congo basin was in the vicinity of the Kouilou-Niari River, located to the north of the present day LCR. Sediment loading and flexural uplift of the interior basin may have played a role in the migration of the Congo outlet, and tectonic activity associated with the Miocene reactivation of the western African margin, appears to have initiated the final capture of the entire drainage by the LCR. This event, resulting in the present day configuration of the main channel of the Congo River, is now generally considered to have occurred at the Miocene-Pliocene transition, and by 2 – 5 myr the high-energy flow regime of the modern LCR was fully established [55–58].

A recent study of two cichlid genera endemic to the LCR [18] estimated that major diversification events in these groups occurred in a roughly similar time frame as the proposed formation of the high-energy, modern LCR system, and to those estimated for LCR *Mastacembelus* in the present study. That study also suggests that the cichlid genus *Steatocranus* colonized the LCR twice, once ca. 4.48 Myr with a second colonization event occurring ca 3.23 Myr. A similar pattern was found for another cichlid genus, *Nanochromis*, which appears to have colonized the LCR considerably later, ca. 2.7 Myr with in a second wave at ca. 1.6 Myr.

### Divergence accompanied by rapid morphological differentiation in *M. brichardi* and *brachyrhinus*

The close phylogenetic relationship between *M. brichardi* and *M. brachyrhinus* is particularly striking given their extremely divergent phenotypes (Figs. 1 and 2). These taxa are reciprocally monophyletic and differentiated from their closest LT relatives, *M. albomaculatus* and *M. tanganicae,* at mitochondrial loci (Additional file 3: Figure S1). However, lack of phylogenetic resolution at nuclear loci between these four taxa likely results from incomplete lineage sorting (ILS) due to relatively recent diversification. Though one individual of *albomaculatus* (SA76223) groups with *brachyrhinus* and *brichardi* in the concatenated tree (Fig. 3), support values are low and the same individual groups with other *albomaculatus* specimens in the mitochondrial tree with high support, suggesting ILS as a possible cause of the discordance. We note, however, that [22] and [26] report the presence of specimens morphologically intermediate between *M. brachyrhinus* and *M. brichardi* and regarded these as hybrids. Those specimens were collected at a single site in the region of Bulu in the middle reach of the LCR, at the time the only know locality where the two species occurred in sympatry. Subsequent collections in the LCR indicate that the two species in fact occur in sympatry at multiple sites along the lower and middle reaches of the LCR as far upstream as the region of Pioka at the border of RC and DRC, above which no specimens of *M. brachyrhinus* have yet been found. In none of these collections have we found evidence of morphological intermediacy, and our molecular data provide no strong support for introgression between the two species. While biogeographic distribution and mtDNA indicate that *brichardi* and *brachyrhinus* are sister species that diverged from each other in the LCR, further studies using additional nuclear markers are needed to disentangle the evolutionary history of this interesting and phenotypically diverse clade, including its LT relatives.

### Genetic differentiation and biogeography within LCR clades

Our molecular analyses demonstrate that three of the *Mastacembelus* species endemic to the LCR region, *M. crassus*, *M. aviceps* and *M. simbi* together represent a well-supported clade, and morphological examination suggests that *M. latens* is also a member of this lineage (unpublished data). However, separate analysis of nuclear and mitochondrial loci indicate that *M. aviceps* and *M. crassus* are reciprocally monophyletic at nuclear but not at mitochondrial loci, suggesting recent introgression. While little is known of the detailed ecology of either species the two have been collected in sympatry and are morphologically, diagnosably distinct, with markedly different facies [4, 22] (Fig. 1). Unfortunately tissues are currently available only from two individuals of *M. aviceps* and additional specimens and genetic markers will be needed to fully resolve the relationship between these two closely related species and to clarify the history of introgression.

Markedly different patterns of population structure and gene flow were observed in *M. simbi* and *M. brichardi*. These two species, both formerly considered strict LRC endemics [3] have recently been collected from a few, isolated deep-water, rocky outcrops just upstream of Pool Malebo [24] thus extending their range a few kilometers beyond Pool Malebo (considered herein as “LCR region”, Fig. 1). Upstream and downstream populations of *M. brichardi* and *M. simbi* occupy similar habitats but are geographically and ecologically separated by Pool Malebo. Pool Malebo (formerly Stanley Pool) is a large (ca. 35 km long, 23 km wide), lake-like expansion of the main stem of the Congo River. Most of the Pool substrate is sand and silt, and extremely shallow (ca. 3 m) although its waters can reach depths of up to 10 m in restricted areas. Upstream (above Pool Malebo) and downstream (below Pool Malebo) populations of *M. brichardi* show no geographic structure (F_ST_ < 0.01, n/s). However, *M. simbi* upstream (above Pool Malebo) and downstream (below Pool Malebo) populations have an *F*_*ST*_ of 0.17 (p < 0.001), despite the lack of any observable morphological differentiation between individuals of either population (unpublished data). Such disparity in population structure, e.g. substantial genetic distance between two locales in *simbi* but not *brichardi*, may result from the relative ages of the clades and timing of colonization as well as the notable difference in size between the two species and their presumed abilities to successfully disperse across the habitat barrier of the Pool.

## Conclusions

Overall, our phylogenetic results demonstrate an instance of striking morphological convergence between two clades of spiny eels in the LCR. Such an example of iterative convergence provides a unique opportunity to address the morphological and genomic basis of adaptation, though progress toward this objective will hinge on the development of more genomic resources for this group. Intriguingly, other taxa within the genus demonstrate the opposite pattern: an extensive degree of cryptic diversity within groups of morphologically indistinguishable specimens. Taxa showing deep divergences within putative species include *M. simbi*, an observation consistent with the findings of [27] for other species in this genus. We speculate that such cryptic diversity results from the generally low dispersal capabilities of many mastacembelids, combined with an apparent paucity of qualitative morphological characteristics in this group, which may hinder accurate morphology-based taxonomy and species diagnosis in some instances. Additional studies that utilize DNA combined with detailed qualitative morphological investigation will be critical to understanding how diversity in this unique clade of fishes has been created and maintained.

## Availability of supporting data

The data sets supporting the results of this article are available as Additional files 1, 2 and 3.

Sequence data are available in GenBank [Accession numbers KT732420 - KT732495; KT732496 - KT732569; KT732570 - KT732646].

## Additional files

Additional file 1: Table S1. Specimens, collection information, and GenBank numbers. (XLSX 46 kb) Additional file 2:
**Supplemental Files: xml files for Bayesian analyses (File 1: concatenated loci; File 2: divergence dating).** (ZIP 49 kb) Additional file 3: Figure S1-S2. Phylogenetic trees constructed using Bayesian inference (BEAST) as described in the text, using (S1) cytochrome b and (S2) concatenated nuclear markers. (ZIP 1811 kb)

## References

[1] Winemiller KO, McIntyre PB, Castello L, Fluet-Chouinard E, Giarrizzo T, Nam S, et al. Hydropower expansion in the Amazon, Congo and Mekong - a looming threat to global biodiversity. Science. In revision. 10.1126/science.aac7082 26744397

[2] Stiassny M, Brummett R, Harrison I, Monsembula R, Mamonekene V, Darwall W, Smith K, Allen D, Holland R, Harrison I, Brooks E (2011). The status and distribution of the freshwater fishes of Central Africa. The diversity of life in African freshwaters: underwater, under threat An analysis of the status and distribution of freshwater species throughout mainland Africa.

[3] Lowenstein JH, Osmundson TW, Becker S, Hanner R, Stiassny ML (2011). Incorporating DNA barcodes into a multi-year inventory of the fishes of the hyperdiverse Lower Congo River, with a multi-gene performance assessment of the genus Labeo as a case study. Mitochondrial DNA.

[4] Vreven EJ, Stiassny ML (2009). Mastacembelus simbi, a new dwarf spiny eel (Synbranchiformes: Mastacembelidae) from the lower Congo River. Ichthyological Exploration of Freshwaters.

[5] Grant PR, Grant BR, Markert JA, Keller LF, Petren K (2004). Convergent evolution of Darwin’s finches caused by introgressive hybridization and selection. Evolution.

[6] Steiner CC, Römpler H, Boettger LM, Schöneberg T, Hoekstra HE (2009). The genetic basis of phenotypic convergence in beach mice: similar pigment patterns but different genes. Mol Biol Evol.

[7] Marchinko KB (2009). Predation’s role in repeated phenotypic and genetic divergence of armor in threespine stickleback. Evolution.

[8] Gleiss AC, Jorgensen SJ, Liebsch N, Sala JE, Norman B, Hays GC, Quintana F, Grundy E, Campagna C, Trites AW (2011). Convergent evolution in locomotory patterns of flying and swimming animals. Nat Commun.

[9] Darwin C (1859). On the origin of species by means of natural selection, or.

[10] Lefébure T, Douady C, Gouy M, Trontelj P, Briolay J, Gibert J (2006). Phylogeography of a subterranean amphipod reveals cryptic diversity and dynamic evolution in extreme environments. Mol Ecol.

[11] Pipan T, Culver DC (2012). Convergence and divergence in the subterranean realm: a reassessment. Biol J Linn Soc.

[12] Rosenblum EB (2006). Convergent evolution and divergent selection: lizards at the White Sands ecotone. Am Nat.

[13] Manceau M, Domingues VS, Linnen CR, Rosenblum EB, Hoekstra HE (2010). Convergence in pigmentation at multiple levels: mutations, genes and function. Philos Trans R Soc Lond B Biol Sci.

[14] Wiens JJ, Chippindale PT, Hillis DM (2003). When are phylogenetic analyses misled by convergence? A case study in Texas cave salamanders. Syst Biol.

[15] Hedges SB, Sibley CG (1994). Molecules vs. morphology in avian evolution: the case of the “pelecaniform” birds. Proc Natl Acad Sci U S A.

[16] McCracken KG, Harshman J, McClellan DA, Afton AD (1999). Data set incongruence and correlated character evolution: an example of functional convergence in the hind-limbs of stifftail diving ducks. Syst Biol.

[17] Snoeks J, Harrison I, Stiassny M, Darwall W, Smith K, Allen D, Holland R, Harrison I, Brooks E (2011). The status and distribution of freshwater fishes. The Diversity of Life in African Freshwaters: Under Water, Under Threat An analysis of the status and distribution of freshwater species throughout mainland Africa.

[18] Schwarzer J, Misof B, Ifuta SN, Schliewen UK (2011). Time and origin of cichlid colonization of the lower Congo rapids. PLoS One.

[19] Jackson P, Oberg K, Gardiner N, Shelton J (2009). Velocity mapping in the lower Congo River: a first look at the unique bathymetry and hydrodynamics of Bulu Reach, West Central Africa. Proceedings of the International Association for Hydraulic Research Congress.

[20] Oberg K, Shelton JM, Gardiner N, Jackson PR (2009). Discharge and Other Hydraulic Measurements for Characterizing the Hydraulics of Lower Congo River, July 2008. Proceedings of the International Association for Hydraulic Research Congress.

[21] Markert JA, Schelly RC, Stiassny ML (2010). Genetic isolation and morphological divergence mediated by high-energy rapids in two cichlid genera from the lower Congo rapids. BMC Evol Biol.

[22] Roberts TR, Stewart DJ (1976). An ecological and systematic survey of fishes in the rapids of the lower Zaire or Congo River. Bull. Mus. Comp. Zool.

[23] Trajano E, Bichuette ME, Kapoor B (2010). Biology of subterranean fishes.

[24] Stiassny ML, Alter SE (2015). Phylogenetics of Teleogramma, a Riverine Clade of African Cichlid Fishes, with a Description of the Deepwater Molluskivore-Teleogramma obamaorum-from the Lower Reaches of the Middle Congo River. Am. Mus. Novit.

[25] Eschmeyer, WN, Fong, JD. "Species by family/subfamily." Catalog of Fishes electronic version. See http://research.calacademy.org/research/ichthyology/catalog/SpeciesByFamily.asp (accessed 20 March 2015) (2015).

[26] Vreven E. A systematic revision of the African spiny-eels (Mastacembelidae, Synbranchiformes). Leuven, Belgium: Katholieke Universiteit Leuven; 2001.

[27] Brown KJ, Rüber L, Bills R, Day JJ (2010). Mastacembelid eels support Lake Tanganyika as an evolutionary hotspot of diversification. BMC Evol Biol.

[28] Travers RA (1984). A review of the Mastacembeloidei, a suborder of synbranchiform teleost fishes.

[29] Vreven E (2005). Mastacembelidae (Teleostei; Synbranchiformes) subfamily division and African generic division: an evaluation. J Nat Hist.

[30] Nickum JG (2004). Guidelines for the use of fishes in research.

[31] San Mauro D, Gower DJ, Oommen OV, Wilkinson M, Zardoya R (2004). Phylogeny of caecilian amphibians (Gymnophiona) based on complete mitochondrial genomes and nuclear RAG1. Mol Phylogenet Evol.

[32] Chow S, Hazama K (1998). Universal PCR primers for S7 ribosomal protein gene introns in fish. Mol Ecol.

[33] Near TJ, Bolnick DI, Wainwright PC (2005). Fossil calibrations and molecular divergence time estimates in centrarchid fishes (Teleostei: Centrarchidae). Evolution.

[34] Edgar RC (2004). MUSCLE: multiple sequence alignment with high accuracy and high throughput. Nucleic Acids Res.

[35] Excoffier L, Laval G, Schneider S (2005). Arlequin (version 3.0): an integrated software package for population genetics data analysis. Evol. Bioinformatics Online.

[36] Stamatakis A (2014). RAxML version 8: a tool for phylogenetic analysis and post-analysis of large phylogenies. Bioinformatics.

[37] Bouckaert R, Heled J, Kühnert D, Vaughan T, Wu C-H, Xie D, Suchard MA, Rambaut A, Drummond AJ (2014). BEAST 2: a software platform for Bayesian evolutionary analysis. PLoS Comput Biol.

[38] Heled J, Drummond AJ (2010). Bayesian inference of species trees from multilocus data. Mol Biol Evol.

[39] Rambaut A, Drummond A. Tracer v1. 4. In*.*; 2007.

[40] Van Steenberge M, Vreven E, Snoeks J (2014). The fishes of the Upper Luapula area (Congo basin): a fauna of mixed origin. Ichthyological Exploration of Freshwaters.

[41] Betancur-R R, Broughton RE, Wiley EO, Carpenter K, López JA, Li C, et al. The tree of life and a new classification of bony fishes. PLoS Currents. 2013;5. 10.1371/currents.tol.53ba26640df0ccaee75bb165c8c26288 PMC3644299 23653398

[42] Murray AM (2006). A new channid (Teleostei: Channiformes) from the Eocene and Oligocene of Egypt. J. Paleontol.

[43] Near TJ, Eytan RI, Dornburg A, Kuhn KL, Moore JA, Davis MP, Wainwright PC, Friedman M, Smith WL (2012). Resolution of ray-finned fish phylogeny and timing of diversification. Proc Natl Acad Sci.

[44] Drummond AJ, Suchard MA, Xie D, Rambaut A (2012). Bayesian phylogenetics with BEAUti and the BEAST 1.7. Mol Biol Evol.

[45] Salzburger W, Meyer A, Baric S, Verheyen E, Sturmbauer C (2002). Phylogeny of the Lake Tanganyika cichlid species flock and its relationship to the Central and East African haplochromine cichlid fish faunas. Syst Biol.

[46] Day JJ, Santini S, Garcia-Moreno J (2007). Phylogenetic relationships of the Lake Tanganyika cichlid tribe Lamprologini: the story from mitochondrial DNA. Mol Phylogenet Evol.

[47] Lévêque C (1997). Introductions de nouvelles espèces de poissons dans les eaux douces tropicales: objectifs et conséquences. Bull. Fr. Peche Piscic.

[48] Skelton P. A Bew Species of Mastacembelus(Pisces, Mastacembelidae) from the Upper Zambezi River, with a Discussion of the Taxonomy of the Genus from this System. Annals of the Cape Provincial Museum(Natural History). 1976;11(6):103–16.

[49] Pinton A, Agnèse J-F, Paugy D, Otero O (2013). A large-scale phylogeny of Synodontis (Mochokidae, Siluriformes) reveals the influence of geological events on continental diversity during the Cenozoic. Mol Phylogenet Evol.

[50] Katongo C, Koblmüller S, Duftner N, Mumba L, Sturmbauer C (2007). Evolutionary history and biogeographic affinities of the serranochromine cichlids in Zambian rivers. Mol Phylogenet Evol.

[51] Goodier SA, Cotterill FP, O’Ryan C, Skelton PH, de Wit MJ (2011). Cryptic diversity of African tigerfish (Genus Hydrocynus) reveals palaeogeographic signatures of linked Neogene geotectonic events. PLoS One.

[52] Day JJ, Peart CR, Brown KJ, Friel JP, Bills R, Moritz T (2013). Continental diversification of an African catfish radiation (Mochokidae: Synodontis). Syst Biol.

[53] Cohen AS, Soreghan MJ, Scholz CA (1993). Estimating the age of formation of lakes: an example from Lake Tanganyika, East African Rift system. Geology.

[54] Weiss JD, Cotterill FP, Schliewen UK (2015). Lake Tanganyika—A‘Melting Pot’of Ancient and Young Cichlid Lineages (Teleostei: Cichlidae)?. PLoS One.

[55] Flügel TJ, Eckardt FD, Cotterill FP. The present day drainage patterns of the Congo river system and their Neogene evolution. In: Geology and Resource Potential of the Congo Basin. Leuven, Belgium: Springer; 2015: 315–337

[56] Giresse P (2005). Mesozoic–Cenozoic history of the Congo basin. J. Afr. Earth Sci.

[57] Stankiewicz J, de Wit MJ (2006). A proposed drainage evolution model for Central Africa—Did the Congo flow east?. J. Afr. Earth Sci.

[58] Anka Z, Séranne M, Lopez M, Scheck-Wenderoth M, Savoye B (2009). The long-term evolution of the Congo deep-sea fan: A basin-wide view of the interaction between a giant submarine fan and a mature passive margin (ZaiAngo project). Tectonophysics.

