# Supplementary figures and images for: Molecular phylogenetics reveals convergent evolution in lower Congo River spiny eels
Source: BMC Evol Biol. 2015 Oct 15;15:224. doi: 10.1186/s12862-015-0507-x (PMC4608218; doi:10.1186/s12862-015-0507-x)

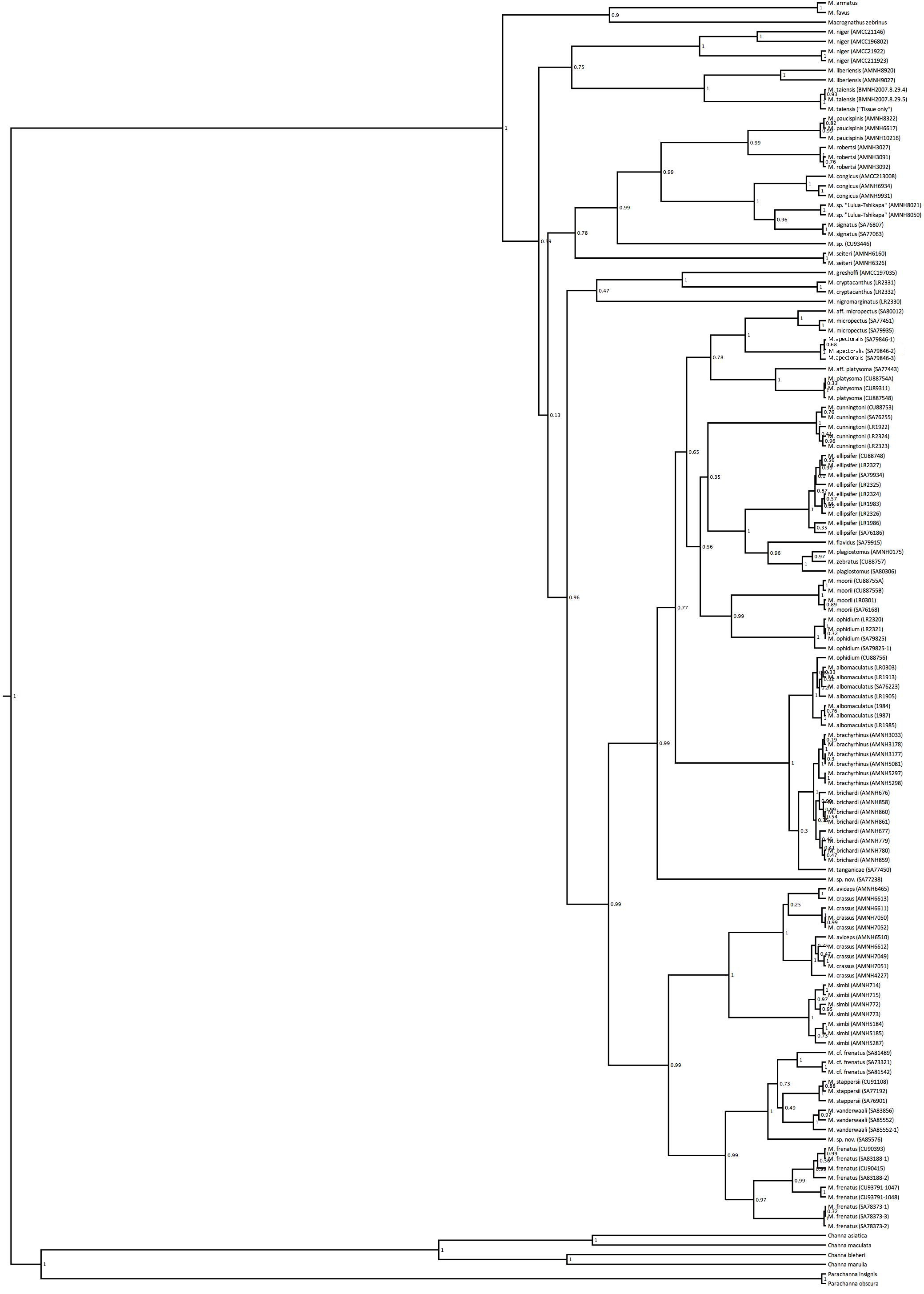

Supplement: Additional file 3: Figure S1-S2. — Phylogenetic trees constructed using Bayesian inference (BEAST) as described in the text, using (S1) cytochrome b and (S2) concatenated nuclear markers. (ZIP 1811 kb) [file 12862_2015_507_MOESM3_ESM.zip › additional file 3/Figure S1 cyt b tree.tif]

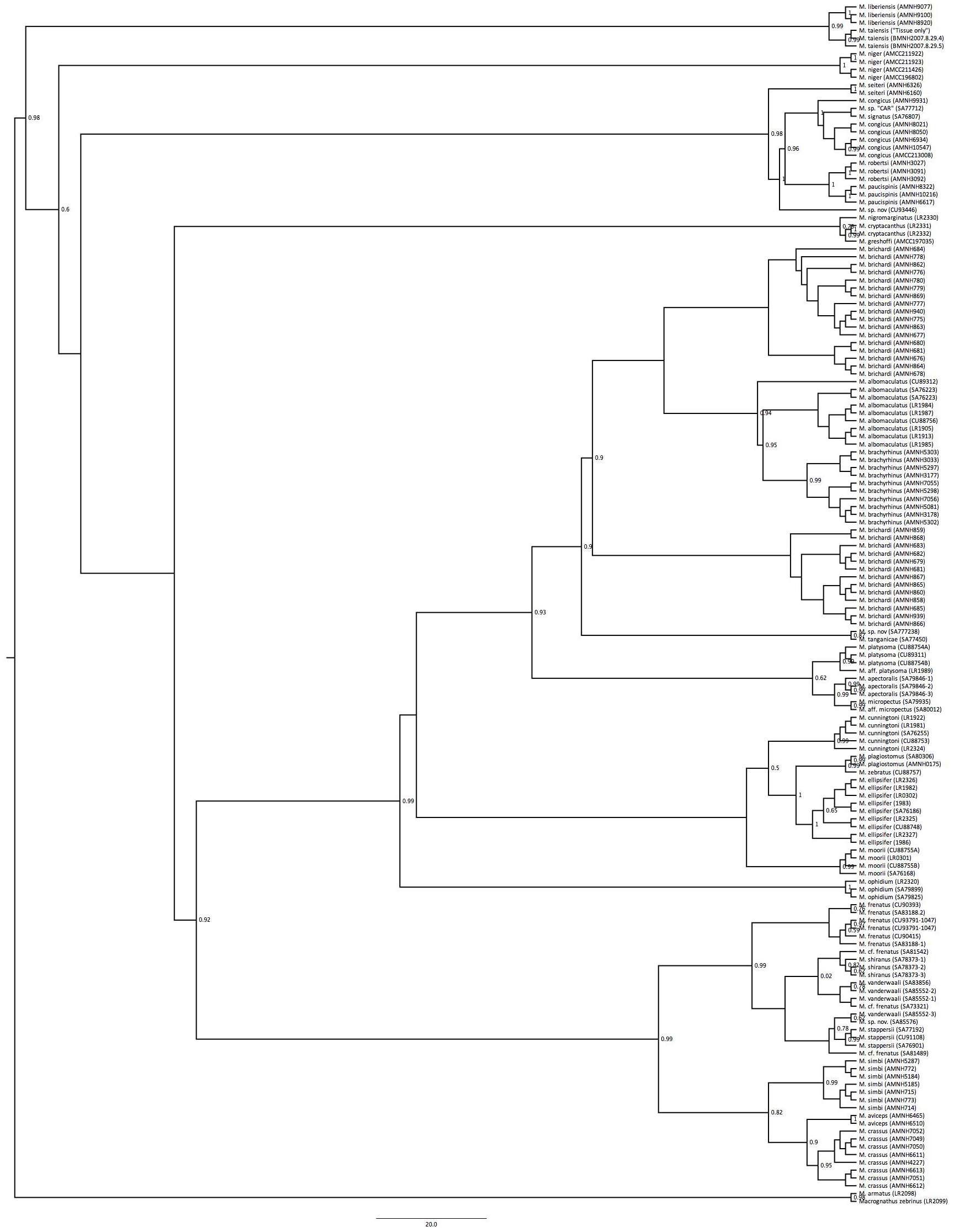

Supplement: Additional file 3: Figure S1-S2. — Phylogenetic trees constructed using Bayesian inference (BEAST) as described in the text, using (S1) cytochrome b and (S2) concatenated nuclear markers. (ZIP 1811 kb) [file 12862_2015_507_MOESM3_ESM.zip › additional file 3/Figure S2 nuclear concatenated.tif]
